# Strengthening the link between mitophagy and Parkinson’s disease

**DOI:** 10.1093/brain/awac405

**Published:** 2022-11-02

**Authors:** Ian G Ganley

**Affiliations:** MRC Protein Phosphorylation and Ubiquitylation Unit, University of Dundee, Dundee, UK

## Abstract

This scientific commentary refers to ‘Regulation of mitophagy by the NSL complex underlies genetic risk for Parkinson's disease at 16q11.2 and MAPT H1 loci’ by Soutar *et al*. (https://doi.org/10.1093/brain/awac325); and ‘DJ-1 is an essential downstream mediator in PINK1/parkin-dependent mitophagy’ by Imberechts *et al.* (https://doi.org/10.1093/brain/awac313).

**This scientific commentary refers to ‘Regulation of mitophagy by the NSL complex underlies genetic risk for Parkinson's disease at 16q11.2 and MAPT H1 loci’ by Soutar *et al*. (https://doi.org/10.1093/brain/awac325); and ‘DJ-1 is an essential downstream mediator in PINK1/parkin-dependent mitophagy’ by Imberechts *et al.* (https://doi.org/10.1093/brain/awac313).**

There is great effort involved in trying to understand the molecular details of Parkinson’s disease, especially given that it is currently incurable.^1^ While the majority of cases are sporadic, a small percentage (10–15%) are hereditary and it is through identification and study of the genes involved in these instances that a light is being shone on the molecular nature of this disease.

Though mitochondrial dysfunction is known to be associated with Parkinson’s disease, it was a landmark study on one particular gene, *PRKN*/PARK2, that illuminated how dysfunctional mitochondria might accumulate.^2^ In this study, the Youle laboratory found that the E3 ligase parkin, encoded by *PRKN*, is recruited to damaged mitochondria to stimulate mitophagy. Mitophagy is a form of autophagy, whereby a mitochondrion is engulfed by a double-membraned organelle termed an autophagosome, which is then degraded following fusion of the autophagosome with a lysosome (Fig. 1).^3^ Following the parkin-mitophagy link, multiple groups demonstrated that *PINK1*/*PARK6*—a Parkinson’s disease gene encoding a kinase that is thought to operate in the same pathway as parkin—functions upstream to sense mitochondrial damage (via membrane depolarization) and in turn activates parkin to cause mitochondrial ubiquitylation and mitophagy (Fig. 1).^3^ These studies have given rise to the hypothesis that mitophagy failure leads to the accumulation of damaged and dysfunctional mitochondria observed in Parkinson’s disease, which in turn leads to further cellular damage, inflammation and ultimately cell death—in particular, in the highly energetic dopaminergic neurons that characteristically degenerate in the disorder.

**Figure 1:**
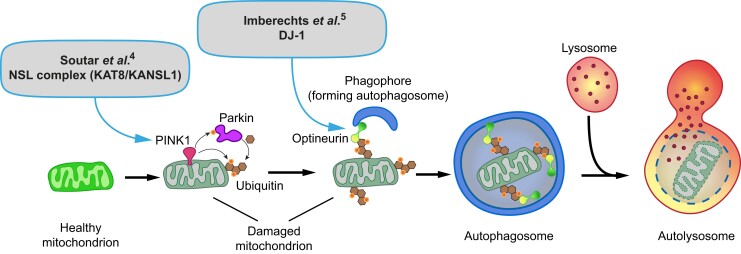
**Basic overview of PINK1/parkin-dependent mitophagy and the newly uncovered roles of the NSL complex and DJ-1**. Mitochondrial damage and depolarization lead to stabilization of the PINK1 protein kinase on the outer mitochondrial membrane. This in turn leads to recruitment and activation of parkin, via phosphorylation of parkin and ubiquitin. This results in a feed forward activation of parkin and enhanced parkin-mediated ubiquitylation of mitochondrial outer membrane proteins. These ubiquitylation events lead to recruitment of optineurin, an autophagy receptor protein that binds directly to ubiquitin and the autophagy initiation machinery. This allows growth of the phagophore, which engulfs the damaged mitochondrion. Once the autophagosome has formed, it traffics to, and fuses with, a lysosome to form the digestive hybrid organelle termed an autolysosome. New work, discussed in the main text,^4,5^ places the NSL complex early in the pathway at the level of *PINK1* gene expression; as well as DJ-1 at a later stage that is critical for optineurin recruitment.

Studying familial cases of Parkinson’s disease has led to the identification of causative mutations in at least 22 genes, and large-scale genome-wide association (GWAS) studies have identified numerous Parkinson’s disease risk loci.^1^ Many of these genes encode proteins thought to influence mitochondrial and lysosomal function (either directly or indirectly), but whether they affect mitophagy is largely unknown, as is whether impaired mitophagy definitely leads to Parkinson’s disease. However, two new papers published in *Brain* now provide compelling evidence that mitophagy is a common mechanism involved in Parkinson’s disease pathology.^4,5^

In the first study, Soutar and colleagues^4^ in the group of Plun-Favreau delved into the extensive pool of GWAS data, with the reasoning that some of these genes may also be involved in PINK1/parkin-dependent mitophagy. Using an innovative combination of bioinformatic techniques, the authors identified 31 open reading frames that displayed a high potential to be *bone fide* Parkinson’s disease risk genes. To determine if these candidates were involved in the regulation of mitophagy, the authors developed a high content microscopy-based siRNA screening assay to monitor PINK1 activity. Here, the levels of the PINK1 substrate phospho-ubiquitin, as well as its localization to mitochondria, were determined following siRNA treatment in neuroblastoma cells that overexpress parkin (used to enhance activity of the mitophagy pathway). Among the candidate genes, only *KAT8* siRNA (as well as the *PINK1* siRNA control) resulted in a significant reduction of phospho-ubiquitin. KAT8 is a lysine acetyl transferase and catalytic subunit of the NSL (non-specific lethal) complex, which regulates acetylation of histone H4. This results in chromatin decompaction and facilitates gene expression in many crucial cellular processes.^6^ Interestingly and confirming specificity, depletion of other lysine acetyl transferases did not alter PINK1 activity but importantly, depletion of other NSL subunits did. Of relevance, KANSL1—a KAT8 interacting subunit of the NSL—was previously identified in a Parkinson’s disease GWAS study.^7^ As might be expected given the loss of PINK1 activity, depletion of KAT8 and KANSL1 impaired downstream parkin activation, its mitochondrial recruitment and mitophagy in response to depolarization. Taken together, this suggests the NSL complex, and in particular KAT8 and KANSL1, are Parkinson’s disease-relevant proteins.

How then do KAT8 and KANSL1 regulate PINK1 mechanistically? Given the known epigenetic remodelling functions of the NSL complex, a prime mechanism is likely through *PINK1* gene expression, and this appears to be the case: both PINK1 mRNA and protein levels were reduced upon depletion of KAT8 and KANSL1. The authors were able to confirm the results in other cell types, including human induced pluripotent stem cell (iPSC)-derived neurons. Physiological relevance for this pathway was further established in a fly model system, whereby depletion of *Drosophila KAT8* and *KANSL1* genes (*mof* and *nsl1*, respectively) resulted in impaired motor function, reduced lifespan, and in the case of *nsl1*, dopaminergic neuron degeneration. Taken together, this very exciting body of work adds further to our understanding of PINK1/parkin-dependent mitophagy at a very early step in the pathway. Importantly, this works stems from a study on Parkinson’s disease risk loci that suggests this step is very relevant for disease pathology. Of course, questions remain: if gene expression is the main mechanism of regulation, is *PINK1* the only Parkinson’s disease-relevant gene disrupted? Is catalytic activity of KAT8 important here and if so, might other proteins be acetylated to regulate mitochondrial function in a more direct manner? Importantly, could enhancing this pathway prove beneficial therapeutically?

In the second study, Imberechts and colleagues^5^ in Wim Vandenberghe’s group took a more targeted approach and specifically analysed the function of DJ-1 in mitophagy. The protein DJ-1, encoded by the *PARK7* gene, is mutated in rare forms of Parkinson’s disease. The functions of DJ-1 are still not clear, though it has links to mitochondria where it can act as a redox sensor and its loss in cells can result in mitochondrial dysfunction.^1^ In this investigation, the authors directly examined PINK1/parkin-dependent mitophagy in the presence and absence of functional DJ-1. Using skin-derived fibroblasts from a Parkinson’s disease patient with a homozygous loss-of-function mutation in *PARK7*, they found that mitophagy was impaired following induction by valinomycin-mediated mitochondrial depolarization. This could also be mimicked in fibroblasts from healthy individuals following siRNA-mediated DJ-1 depletion. Interestingly, this did not appear to require the redox sensing functions of DJ-1, given that mutation of the oxidation-sensitive cysteine at position 106 was able to rescue the mitophagy defect as well as wild-type protein. Impaired mitophagy was also observed when patient fibroblasts were reprogrammed to iPSCs and differentiated into neurons.

In contrast to the study by Soutar and colleagues,^4^ when the authors looked at the stages of mitophagy that were impaired, they found no effect on the activation of PINK1 and parkin, nor the mitochondrial deposition of ubiquitin and phosphoubiquitin. This suggests that DJ-1 functions downstream of PINK1/parkin mitophagy initiation. A key function of parkin-mediated mitochondrial ubiquitylation is thought to be in recruiting the autophagic machinery, whereby ubiquitin binds directly to members of the sequestosome-like receptor (SLR) family of proteins (Fig. 1).

SLRs can simultaneously bind to ubiquitin and autophagosome initiating proteins including FIP200 and the ATG8s,^3^ and PINK1/parkin-dependent mitophagy is dependent on the SLRs NDP52 and optineurin.^8^ In neuronal tissue, optineurin is the relevant SLR regulating mitophagy,^8^ and Imberechts and colleagues^5^ found that loss of DJ-1 prevented recruitment of optineurin to ubiquitylated mitochondria and hence initiation of autophagosome formation. Using elegant proximity ligation assays as well as co-immunoprecipitation, they showed that these two proteins are likely in a complex together and co-translocate to mitochondria following PINK1/parkin activation. Indeed, artificially targeting DJ-1 to mitochondria is sufficient to also recruit optineurin (but it is not clear if this is sufficient for mitophagy). The authors’ data suggests it is this DJ-1:optineurin complex that is important for mitochondrial recruitment.

These findings clearly put DJ-1 into the PINK1/parkin pathway at a critical point involving autophagosome initiation at mitochondria. However, how exactly DJ-1 regulates optineurin function and recruitment to mitochondria is not yet clear, especially as optineurin is thought to bind directly to ubiquitin conjugated by parkin on the mitochondrial outer membrane. It is also interesting to note that the authors found no role for NDP52 in their fibroblast mitophagy model system, even though it is expressed at detectable levels (in comparison to neurons). Given that redundancy between NDP52 and optineurin exists in some cell types,^8^ further elucidation of the relationship between DJ-1, optineurin and NDP-52 may help to design therapeutic approaches that specifically target neuronal mitophagy.

As mentioned, mitochondrial dysfunction is a key hallmark of Parkinson’s disease pathology but whether this is caused by compromised mitophagy is far from clear. Given the diverse nature of Parkinson’s disease genes, as well as environmental factors linked to sporadic forms of the disease, it is likely that there are multiple pathways and mechanisms leading to mitochondrial impairment. It is important to note that non-mitophagy functions have also been ascribed to PINK1 and parkin,^9^ and it is possible that these are key for Parkinson’s disease.

In addition, mitophagy can occur independently of PINK1 and parkin.^3^ Indeed, recent work has shown that the most common Parkinson’s disease mutation found to date, LRRK2 G2019S, impairs mitophagy in dopaminergic neurons and microglia within mouse brain, independently of PINK1.^10^ Thus, disruption of multiple mitophagy pathways may be relevant to Parkinson’s disease. Regardless, even if impaired mitophagy is not a significant driver of mitochondrial dysfunction, it is still likely a therapeutically advantageous pathway, if enhanced mitophagy can help remove and recycle these deleterious mitochondria. Therefore, these two studies not only offer new insights into the mechanisms regulating PINK1/parkin-dependent mitophagy but also reveal how disruption of distinct steps in this pathway could lead to Parkinson’s disease. Excitingly, they also suggest new candidates for therapeutic intervention.
